# Interaction of a traditional Chinese Medicine (PHY906) and CPT-11 on the inflammatory process in the tumor microenvironment

**DOI:** 10.1186/1755-8794-4-38

**Published:** 2011-05-11

**Authors:** Ena Wang, Scott Bussom, Jinguo Chen, Courtney Quinn, Davide Bedognetti, Wing Lam, Fulan Guan, Zaoli Jiang, Yichao Mark, Yingdong Zhao, David F Stroncek, Jeffrey White, Francesco M Marincola, Yung-Chi Cheng

**Affiliations:** 1 Infectious Disease and Immunogenetics Section (IDIS), Department of Transfusion Medicine, Clinical Center and trans-NIH Center for Human Immunology (CHI), National Institutes of Health, Bethesda, Maryland, 20892, USA; 2 Department of Pharmacology, Yale University School of Medicine, New Haven, Connecticut, 06520, USA; 3 Department of Oncology, Biology and Genetics and Department of Internal Medicine, University of Genoa and National Cancer Research Institute of Genoa, Largo Rosanna Benzi 16132 Genoa, Italy; 4 Division of Cancer Treatment and Diagnosis, National Cancer Institute, National Institutes of Health, Bethesda, Maryland, 20892, USA; 5 Cell Therapy Section, Department of Transfusion Medicine, Clinical Center, National Institutes of Health, Bethesda, Maryland, 20892, USA; 6 Office of Cancer Complementary and Alternative Medicine, Division of Cancer Treatment and Diagnosis, National Cancer Institute, National Institutes of Health, Bethesda, Maryland, 20892, USA

## Abstract

**Background -:**

Traditional Chinese Medicine (TCM) has been used for thousands of years to treat or prevent diseases, including cancer. Good manufacturing practices (GMP) and sophisticated product analysis (PhytomicsQC) to ensure consistency are now available allowing the assessment of its utility. Polychemical Medicines, like TCM, include chemicals with distinct tissue-dependent pharmacodynamic properties that result in tissue-specific bioactivity. Determining the mode of action of these mixtures was previously unsatisfactory; however, information rich RNA microarray technologies now allow for thorough mechanistic studies of the effects complex mixtures. PHY906 is a long used four herb TCM formula employed as an adjuvant to relieve the side effects associated with chemotherapy. Animal studies documented a decrease in global toxicity and an increase in therapeutic effectiveness of chemotherapy when combined with PHY906.

**Methods -:**

Using a systems biology approach, we studied tumor tissue to identify reasons for the enhancement of the antitumor effect of CPT-11 (CPT-11) by PHY906 in a well-characterized pre-clinical model; the administration of PHY906 and CPT-11 to female BDF-1 mice bearing subcutaneous Colon 38 tumors.

**Results -:**

We observed that 1) individually PHY906 and CPT-11 induce distinct alterations in tumor, liver and spleen; 2) PHY906 alone predominantly induces repression of transcription and immune-suppression in tumors; 3) these effects are reverted in the presence of CPT-11, with prevalent induction of pro-apoptotic and pro-inflammatory pathways that may favor tumor rejection.

**Conclusions -:**

PHY906 together with CPT-11 triggers unique changes not activated by each one alone suggesting that the combination creates a unique tissue-specific response.

## Background

Although traditional Chinese medicine (TCM) has been applied for thousands of years in Asia, its recognition by Western countries rests upon objective documentation of its value. To date, no systematic evaluation of TCM has provided conclusive proof of efficacy, though several studies suggest beneficial applications in certain patient populations [1]. In cancer patients, TCM has been applied predominantly to control cancer-associated symptoms or decrease treatment-related toxicity. However, it was observed that some herbal products display anticancer properties [2]. Most TCM products currently used in cancer therapy display a broad range of biological effects including pro-apoptotic activity, inhibition of angiogenesis and boosting of immune response. For instance, it was recently observed that a significant proportion of ginseng's biological activity *in vivo *is exerted through modulation of innate immunity [3].

PHY906 is a TCM formulation that has been used for the last 1,800 years to treat distressing conditions of the gastrointestinal tract. Several preclinical animal models tested the ability of PHY906 to increase the therapeutic window of chemotherapy by decreasing its gastrointestinal side effects. Recently, we documented strong protective effects by PHY906 on chemotherapy-induced intestinal toxicity [4,5]. Moreover, pre-clinical and early-phase clinical trials of PHY906 in combination with chemotherapy in patients with advanced hepatocellular carcinoma [6], pancreatic cancer and other gastrointestinal malignancies [7] have yielded promising results.

To date, no experimentally-validated hypothesis about the adjuvant anti-tumor mechanisms of PHY906 given during chemotherapy has been proposed. Extensive biochemical characterization and standardization of PHY906, which includes a mixture of four herbs: *Glycyrrhiza uralensis *Fisch (G), *Paeonia lactiflora *Pall (P), *Scutelleria baicalensis *Georgi (S), and *Ziziphus jujuba *Mill (Z) under GMP conditions, using Phytomics technology indicates that the product can be prepared with a high degree of consistency [8] greatly improving the interpretability of pre-clinical and clinical results. Chemical and biological characterization of PHY906 demonstrated that a large number of bioactive substances are present in the four herbal preparations. Because of the pharmacokinetic differences in the processing and creation of structurally different chemicals, and the pharmacodynamic differences of new metabolites vs. parent compounds, the biological effects identified in *vitro *are difficult to ascribe as a relevant anti-cancer mechanism *in vivo *[9]. Moreover, none of the herbs display alone *in vivo *the effectiveness displayed by their combination. This complexity has hampered the design of mechanistic studies evaluating specific biological pathways in the context of the cumulative effects of PHY906, particularly when its effects are evaluated *in vivo *where different active components have the opportunity to interact with cancer and normal host's cells simultaneously. Therefore, a first effort toward the understanding of the mechanisms of action of PHY906 should frame the overall effects of the herb *in vivo *using global transcriptional profiling. Thus, we scanned alterations of the transcriptional program induced by PHY906 following its administration as a single agent and in combination with Irinotecan (CPT-11) in a well-characterized pre-clinical model in which both agents were administered to female BDF-1 mice bearing subcutaneous Colon 38 tumor implants. Its effects were compared with those observable in normal tissues such as autologous liver and spleen. The study demonstrated that PHY906 or CPT-11 alone induces significant transcriptional changes in the tumor and in normal tissues that are exquisitely tissue-specific. PHY906 significantly amplifies the effects of CPT-11 in the tumor tissue. Most importantly, PHY906 together with CPT-11 triggers unique changes not triggered by each one alone and this may explain the enhanced anti-tumor activity of the combination.

## Materials and methods

### Animal Model

Murine Colon 38 cells were transplanted subcutaneously into four- to six-week-old female BDF1 mice (Charles River Laboratories, Wilmington, MA) as previously described [4]. After 10 to 14 days, mice with tumor sizes of 150-300 mm^3 ^were selected. PHY906 was given orally over 72 hours (500 mg/kg) while CPT-11 (360 mg/kg) was administered intra-peritoneally on Day 0. In the combination group, the first dose of PHY906 was given 30 minutes prior to CPT-11 administration. Mice (BDF1 bearing Colon 38 tumors) were terminated by cervical dislocation 72 hours after initiation of drug treatment. Tumor tissue was removed and divided and either frozen for total RNA isolation or placed in 10% neutral buffered formalin for histological analysis. All animal experiments were carried out in accordance with an approved Yale University Institutional Animal Care and Use Committee (IACUC) protocol. Murine Colon 38 cell lines were provided by Dr. Giuseppe Pizzorno, Ph.D., Pharm.D (Translational Science, Nevada Cancer Institute, USA).

### Gene expression microarray analysis

Total RNA was extracted from mouse tissues using TRIzol^® ^(Invitrogen) and 3 μg were amplified by in vitro transcription [10]. Pooled total RNA from mixed normal mouse tissues was used as universal reference. Both test and reference RNA were labeled respectively with Cy5 and Cy3 using ULS aRNA Fluorescent Labeling kit (Kreatech, Netherlands) and co-hybridized to a 36K mouse oligonucleotide arrays (Operon, Version 4.0 Mouse Genome Oligo Set containing 35,852 probes representing approximately 25,000 genes). Hybridization was carried out overnight at 42°C and the microarray slides were scanned by GenePix Pro 4.0 (Axon, Sunnyvale, CA).

### Microarray data analysis and statistics

The resulting data files were analyzed using BRBArray Tools [11]. Raw data were background subtracted. The complete experimental data set including 111 samples from mouse tumor, liver and spleen was filtered retaining transcripts with at least 80% spot presence with spot size requirement >20 μM, and intensity in at least one channel > 200 and override the other channel to 200 to avoid high ratio artifact. Intensity ratios above 64 were truncated. Data were normalized using median normalization across the array. This filter selected 18,549 transcripts that were used as the master data set for subsequent analyses. A high stringency comparison (significance p-value cutoff < 0.001) was used to identify transcripts with a high likelihood of differential expression among experimental groups and a low stringency gene enrichment (cutoff p-value < 0.05) was used for pathway analysis. For all experiments, multivariate (global) permutation test (*pt *= 10,000 *pt*s) was applied to test whether the number of transcripts obtained in each analysis was above the likelihood expected by chance. Gene functions were congregated into pathways and networks using Ingenuity Pathways Analysis (IPA) software (v7.6). Canonical pathways enriched in differentially expressed transcripts were ranked according their significance (-log p value).

### Real-Time qPCR

For each sample, 0.5 to 1 ug of total RNA was converted into cDNA using M-MLV reverse transcriptase according to manufactures instructions using random primers. All individual primer sets for genes of interest were designed using NCBI primer design on exons junction when possible. Primer pairs were then run through Beacon Designer™ Free qPCR Designer Program http://www.premierbiosoft.com/jsp/marketing/FreeToolLogin.jsp?PID=1 to eliminate primer-dimers that may interfere with assay. Primers were then checked using melt curve analysis for one product and their efficiency determined using iCycler software. Real Time PCR was performed on BioRad-i-Cycler system using Sybr^® ^green as dye and Phusion^® ^DNA Polymerase with the following condition: 98°C 1 min followed by (98°C :10 sec, 60°C :10 sec 72°C :10 sec) X either 38 or 44 cycles. Sample serial dilutions served as source for relative standard curve. Samples (n = 10/group) were run in duplicate. For each sample, the relative copy number for the gene of interest is divided by the relative copy number for B-actin. Final result is presented as relative expression of the gene relative copy number compared to the mean of the same gene in PBS control group. Results calculated using Excel and graphed in GraphPad Prism. Primer sequences are shown in Additional file 1, Table S1

### Immunohistochemistry

Tissues were embedded in OCT, stored in liquid nitrogen and cut into 10 μm sections at -18°C. The tissue sections were fixed in acetone at 4°C for 10 minutes and stored at -80°C. Upon immunohistochemistry staining, sections were post-fixed in methanol with 3% acetic acid for 20 minutes. After two 5 minute washes in PBS, the sections were bathed for 20 minutes in 3% H_2_O_2 _in Tris Buffered Saline with 0.05% Tween-20 followed by incubation for 1 hour in blocking buffer (Gelatin 0.1%, BSA 1%, Goat serum 10% in TBS). Primary antibody F4/80 (abcam #ab16911 rat monoclonal-BM8) was diluted 1:10 in blocking buffer and incubated with the tissue sections overnight at 4°C. After washing 4 times in PBS and TBS at room temperature, rat on mouse HRP-Polymer Kit (#RT517G, BIOCARE) was incubated with the section for 20 minutes. After washing, the HRP polymer that conjugates to the probe was added for 20 minutes followed by DAB development. Sections were then counter stained with hematoxylin.

## Results

### Effect of PHY906 on tumor growth and weight in CPT-11 treated BDF-1 mice bearing colon 38 tumors

**Figure 1:**
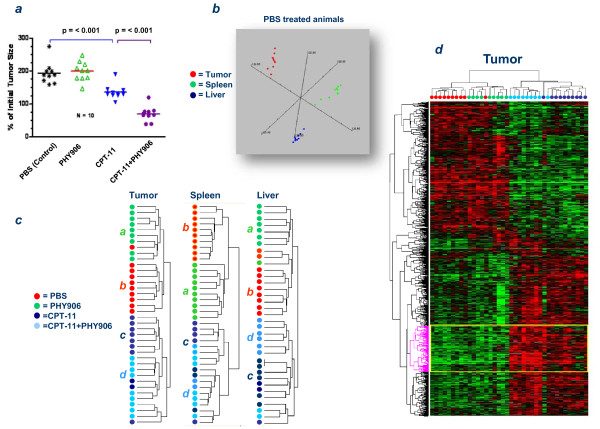
**Effect of PHY906 and/or CPT-11 on tumor, spleen and liver**. (***a***) Effect of PHY906 and/or CPT-11 on tumor size 72 hours after the initiation of treatment. The p-values refer to unpaired Student *t *test between groups. (***b***) Multidimensional scaling (MDS) based on the complete filtered data set including 18,549 transcripts demonstrating in Euclidian space distances among tumor, liver and spleen of animals treated with PBS. (***c***) Unsupervised, self-organizing clustering based on transcripts that passed for each tissue studied a filter requirement of 80% presence and at least one experiment with a ratio above 4 out of 18,549 that originally passed the less stringent filter (experimental clustering based on Kendal's *Tau *regression model, tumor = 2, 635 transcripts, spleen = 2,079 transcripts, liver = 1,059). (***d***) Self-organizing clustering based on 700 genes out of 1,132 tumor genes differentially expressed among the four treatment groups (F test, p-value cutoff < 0.001) that passed a filter of 80% presence and a log ratio ≥ 3 in at least one experiment. Highlighted in yellow is the area of the heat map were enhancement of the CPT-11 effects by the combination CPT-11+PHY906.

Forty BDF-1 mice, each bearing a colon 38 tumor, were divided into the following four treatment groups: Phosphate Buffered Saline (PBS, control), PHY906 (500 mg/Kg, twice a day for 72 hours), CPT-11 (Camptosar^®^, 360 mg/kg), or = PHY906(500 mg/Kg), followed 30 minutes later by a single injection of CPT-11 (360 mg/kg) with continued twice a day dosing of PHY906 (500 mg/Kg) for 72 hours. Tumors were measured 72 hours after treatment (Figure 1a) and then removed. As expected [6], PHY906 enhanced the anti-tumor activity of CPT-11 although alone it had no effect on tumor growth. These early effects resulted in better long term reduction of tumor growth compared to CPT-11 treatment alone up to the 14^th ^day from the beginning of treatment [12]. No differences in animal body weight were observed between the CPT-11 and the CPT-11 plus PHY906 groups.

### Comparison between the tumor, spleen and liver

Transcriptional patterns observed in the 4 treatment cohorts were compared among liver, spleen and tumor samples. In the absence of treatment (PBS cohort), the transcriptional program of the tumors was remarkably different from normal liver and spleen. Multiple dimensional scaling (MDS) based on the complete data set, clearly separated tissues (Figure 1b). Gene enrichment based on an unpaired *t *test at a low cutoff stringency (p-value < 0.05) identified 7,856 transcripts differentially expressed by tumors compared to the two normal tissues combined. Of these genes, 7,348 had an annotated function and, therefore, could be used for Ingenuity Pathway Analysis (IPA). A large number of canonical pathways (104 over a total of 318) were significantly altered (Fisher's exact test threshold p_2_-value < 0.05) in the tumor microenvironment compared to the normal tissues. Most affected cellular growth and proliferation, intra-cellular signaling and cellular stress. Ranking of pathways according to percentage of genes affected identified several immune-related canonical pathways (IL-22, JAK/STAT, IFN, IL-3 and VEGF signaling) within the top 15 confirming that chronic inflammation is associated with the neoplastic process. While pathways predominantly associated with the oncogenic process were enriched with over-expressed transcripts (cleavage of mRNA, polyamine regulation in colon cancer, DNA methylation and protein ubiquitination), canonical pathways associated with immune function were more balanced in their frequency of up- or down-regulated genes.

Because of the disparities among the transcriptional profiles of various tissues, it became obvious that direct cross comparisons of the effects of treatment among different tissues would not be informative; rather indirect comparisons could be made by defining the effects of various treatments in each tissue and subsequently comparing different tissues. A four way Anova (F test, cutoff p-value < 0.001) and univariate *t *tests (cutoff p-value < 0.001 and < 0.05) were performed among (between) treatments for the three tissues to obtain a general estimate of the number of transcripts proportionally affected in each condition (Additional file 2, Table S2). Both CPT-11 and PHY906 affected a larger number of genes in the spleen than in the tumor or liver. In all tissues, differences were observed among treatment groups and PHY906 treatment alone bore significant and independent effects that were statistically significant by permutation (*pt*) test. Moreover, PHY906 significantly altered the effects of CPT-11 in all tissues. There was little overlap among the effects of the 4 treatments on the tumor or other tissues. The patterns that were dominant in the tumor microenvironment (innate immune response and activation of nuclear factor kappa B; NF-*k*B) were minimally affected in the other two organs. Thus, as expected, the effects of either PHY906 and/or CPT-11 are highly tissue specific.

### Analysis of therapy on the tumor tissues

The effect of PHY906 and CPT-11 on tumor tissues was analyzed by comparing the transcriptional program of tumors from mice treated with PBS (n = 10), PHY906 (n = 9), CPT-11 (n = 10) and CPT-11+PHY906 (n = 9). Unsupervised clustering demonstrated preferential grouping of PHY906 treated samples compared with PBS treated samples (Fisher's exact test p_2_-value < 0.001 between sub-cluster ***a ***vs ***b*, **Figure 1c) while a less clear but still significant separation was observed in tumors from animals treated with CPT-11+PHY906 compared to CPT-11 alone (Fisher's exact test p_2_-value = 0.003 between sub-cluster ***c ***vs ***d***). Furthermore, four tumor samples (one from a mouse treated with CPT-11 alone and three from mice treated with the combination) clustered separately from the other CPT-11 treated animals. A multivariate analysis (F test) adopting a < 0.001 p-value cutoff identified 1,132 genes (global *pt *test p-value < 0.001) differentially expressed among the four groups (Table 1). A self-organizing clustering algorithm based on these transcripts confirmed that salient differences were due to the CPT-11 therapy (Figure 1d). Within each sub-cluster, however, there was significant separation between treatment groups (Fisher's exact test p_2_-value < 0.001 for PBS vs PHY906 and for CPT-11 vs. CPT-11+PHY906). Comparison of the CPT-11+PHY906 with the CPT-11 treatment group demonstrated that PHY906 overall enhanced several transcriptional nodes effected by the latter (Figure 1d, **yellow box**).

**Table 1:** Summary of differentially expressed tumor genes among treatment groups.

Tumor Microenvironment	Test	Number of genes < 0.001 and (<0.05 total/annotated)*	Pt p-value for <0.001 cutoff^†^
Four way	F test	**1,132**	< 0.001

PBS vs PHY906	t test	**117/113 **(1,759/1,664)	< 0.001

PBS vs CPT-11	t test	**570/557 **(2,630/2,513)	< 0.001

PBS vs CPT-11+PHY906	t test	**798/774 **(3,347/3,190)	< 0.001

CPT-11 vs CPT-11+PHY906	t test	**93/91 **(1,556/1,477)	< 0.001

Number of sample: PBS = 10; PHY906 = 9; CPT-11 = 10; CPT-11 + PHY906 = 9; Total 38

*Cutoff of p-value < 0.05 was used to select genes for IPA; In parentheses are the number of genes identified at <0.05 level and with an annotated function that could be used for IPA; ^†^although the *pt *test p-value is shown for the <0.001 analyses, identical results were obtained for the lower stringency analyses. Details concerning comparisons among other tissues and treatments are provided in the extended table as an additional file (Additional file 2, **Table S2**).

Note. *Pt *test: permutation test

### Effect of PHY906 on tumors

**Figure 2:**
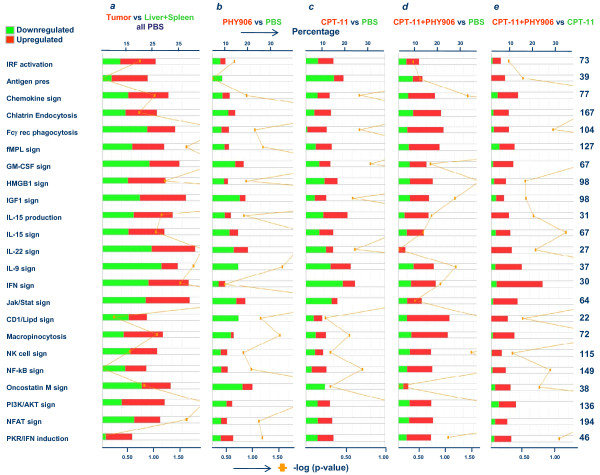
**Effect of PHY906 and/or CPT-11 on tumor specific canonical pathway with immunologic function.** (***a***) Stacked bar chart summarizing the 23 most affected canonical pathways with immunologic function according to IPA based on 7,348 genes with annotated function differentially expressed (*t *test cutoff p-value < 0.001) between colon 38 tumors and the combined liver and spleen database in animals treated with PBS. The same canonical pathways are portrayed looking at the effects on the tumor microenvironment of (***b***) PHY906, CPT-11 (***c***) and CPT-11+PHY906 (***d***). Finally, differences between CPT-11+PHY906 compared to CPT-11 alone are shown (***e***).

A high stringency Student *t *test (cutoff p-value < 0.001) identified 117 genes affected by PHY906 (*pt *test p-value < 0.001(Table 1); of which 113 had an annotated function suitable for IPA. This analysis revealed a self-organizing network that involved predominantly cell to cell contact and movement. A less stringent gene enrichment analysis with a cutoff p-value of < 0.05 yielded 1,759 genes (*pt *test p-value < 0.001) of which 1,664 had an annotated function. The application of IPA to this gene set demonstrated that most transcripts were related to general processes of cellular metabolism, and the majority were down-regulated (Additional file 3, Figure. S3a). In addition, several other canonical pathways related to immune function appeared affected. We, therefore, displayed immune-related canonical pathways side by side comparing their constitutive activation in the tumor microenvironment (compared to the normal tissues, Figure 2a) with their alterations in various treatment groups (Figure 2b-e). PHY906 appeared to suppress the expression of genes belonging to most pathways compared to their constitutive expression in the tumor microenvironment.

### Effect of CPT-11 on tumors

**Figure 3:**
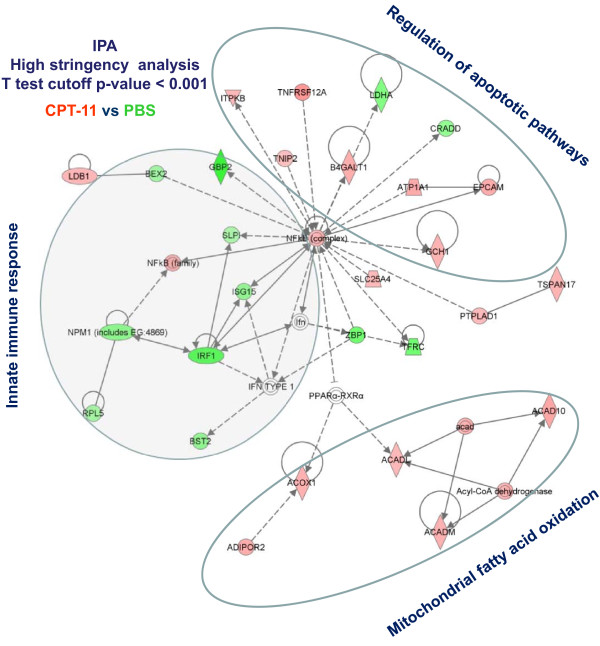
**Immune network predominantly affected by CPT-11 (the analysis performed at high stringency gene selection; t test p-value cutoff < 0.001)**.

**Figure 4:**
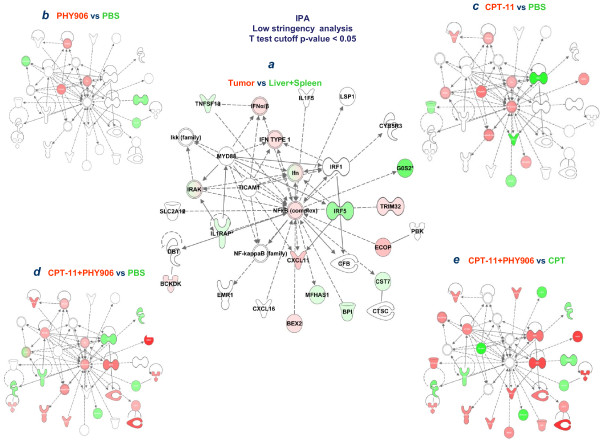
**Immune network predominantly affected by the neoplastic process compared to normal tissues (***a***) and the effect of PHY906 (***b***), CPT-11 (***c***), CPT-11+PHY906 (***d***) or the differential effect of CPT+PHY906 over CPT-11 alone (***e***) (the analysis performed at low stringency for gene enrichment; t test p-value cutoff < 0.05)**.

Class comparison between tumors from mice treated with PBS or CPT-11 identified 570 genes differentially expressed at a Student *t*-test cut-off p-value of < 0.001 (*pt *test p-value < 0.001) of which 557 had an annotated function (Table 1). Congregation of genes into functional networks identified the NF-*k*B family, master regulator of innate immune responses and apoptosis, as the top target of CPT-11 (score 45, focus molecules 28, Figure 3). A dichotomy was noted in the expression of NF-*k*B-dependent genes with a general down-regulation of the transcripts associated with innate immune responses including interferon regulatory factor (IRF)-1 and upregulation of genes regulating apoptosis including mitochondrial fatty acid oxidation [13]. Overlay of the effects of PHY906 alone demonstrated that the herbal extract had no direct effects on this pathway. However, when PHY906 was given in combination with CPT-11, a remarkable reversal of the anti-inflammatory effects induced by CPT-11 was observed. To better illustrate this point, enrichment of genes affected by CPT-11 compared to PBS was performed focusing on the immune regulatory aspects of the treatments (cutoff < 0.05), which identified 2,630 transcripts (2,513 with annotated function for IPA, Additional file 3, Figure. S3b). Compared to the constitutive expression of immune related networks in the tumor microenvironment (Figure 4a), CPT-11 had an ambivalent effect with down regulation of several pro-apoptotic/pro-inflammatory transcripts including IRF-1 and, down-stream of it several interferon stimulated genes (Figure 4c). This is an important observation because IRF-1 is the master regulator of the acute inflammatory switch with powerful pre-inflammatory/pro-apoptotic [14] and anti-angiogenic properties [15]. Side by side comparison of immune pathways between PHY906 and CPT-11 treated animals revealed that the latter had a mixed effect compared with the immune suppressing effects of the former (Figure 2c).

### Effect on tumors of PHY906 when combined to CPT-11

High stringency class comparison between CPT-11+PHY906 and PBS treated animals (*t *test cutoff p-value < 0.001) identified 798 differentially expressed transcripts. Enrichment analysis (threshold p-value of < 0.05) identified 3,347 genes (3,190 with an annotated function) (Table 1). Both data sets suggested that the combination reversed the down-regulation of the expression of genes related to innate immune responses observed with CPT-11 treatment. Comparison of the 15 most affected canonical pathways suggested that the primary differences between combination therapy and CPT-11 alone were quantitative, with more genes being up regulated by the combined treatment (Additional file 3, Figure. S3c). Side by side comparison of immune pathways (Figure 2d) clearly demonstrated that the combination enhanced the expression of pro-inflammatory functions. Thus, PHY906 enhanced the pro-inflammatory effects of the chemotherapy acting in an opposite direction than when given alone (Figure 2b). Overlay of the PHY906 plus CPT-11 effects (Figure 4d) on the CPT-11 modulated NF-*k*B network clearly demonstrated an up regulation of genes associated with NF-*k*B immune function including the activation of the MyD-88/IRF-5 signaling network. Moreover, a diminished suppression of IRF-1 was observed with an average ratio going from 0.3 with CPT-11 vs PBS to 0.64 when CPT-11+PHY906 were compared to PBS.

### PHY906-specific effects during CPT-11 therapy on tumors

To better understand the specific contribution of PHY906 when used in combination with CPT-11, we compared tumor samples from animals treated with CPT-11 to those treated with CPT-11+PHY906 and identified 93 genes that were differentially expressed (*pt *test p-value < 0.001). Enrichment analysis identified 1,556 genes differentially expressed (*pt *test p-value < 0.001, 1,477 annotated) (Table 1). IPA analysis demonstrated that the overlap in the ranking of most affected canonical pathways was limited to pro-inflammatory pathways such as IFN, IL-9 and JAK/STAT signaling, suggesting that the addition of PHY906 to CPT-11 treatment predominantly affected pathways associated with the immune switch from chronic to acute inflammation (Additional file 3, Figure. S3d). These effects could be best appreciated by the side by side comparison (Figure 2e) where PHY906 proportionally enhanced all immune regulatory pathways associated with acute inflammatory processes. The best example was the complete reversal of the expression of genes associated with IFN signaling with up-regulation of the IFN-α receptor, JAK1, STAT-2, IRF-1 and IRF-5.

Combining the analysis of CPT-11+PHY906 vs CPT-11 and CPT-11+PHY906 vs PBS one observes that there is a striking immunologic effect of PHY906 when given in combination with CPT-11 that is an activation of the IRF-5/Myd88 pathways and the reversal of the suppression of the STAT-1/IRF-1 pathways (Figure 4e). This preliminary data suggests that PHY906 works in the context of chemotherapy by enhancing inflammation through IRF-5 while blocking the anti-apoptotic effects likely activated by cancer cells through the suppression of IRF-1 expression [14].

### Validation of immune cell infiltration in tumor by immunohistochemistry

**Figure 5:**
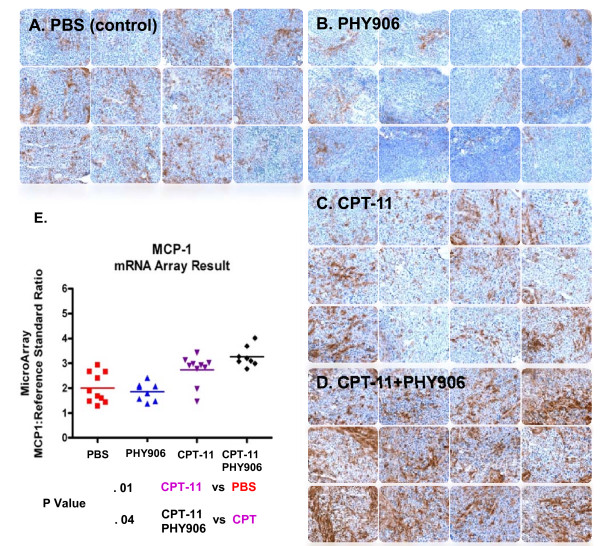
**Macrophage infiltration signatures in tumors treated with PHY906 and/or CPT-11.** Macrophage infiltration in colon 38 tumors treated with PBS (***a***), CPT-11 (***b***), PHY906 (***c***) or Ininotecan+PHY906 (***d***). MCP-1 RNA alteration in tumors induced by various treatments (***e***)

The previous analysis suggested that the colon 38 tumors are characterized by moderate inflammation compared with normal tissues and that this can be modulated by the anti-inflammatory effects of PHY906 alone and the pro-inflammatory effects of CPT-11 and even further by the combination of the two. Thus, we looked at the presence of macrophages in tumors as a general marker of the inflammatory process. In particular we were interested in macrophage infiltration in the combination treatment in which IRF-5 was found strongly up regulated. IRF-5 serves an integral role in the gene induction program activated by Toll-like receptor signaling [16] while playing a potent role as mediator of cell cycle arrest and cell death [17]. Among various pro-inflammatory activities, IRF-5 expressed in mice by activated macrophages can induce the production of several chemokines including CCL-1/I-309, CCL-2/monocyte chemotactic protein 1 (MCP-1), CCL-4/macrophage inflammatory protein 1 β (MIP-1β), CCL-5/Regulated upon Activation, Normal T-cell Expressed, and Secreted (RANTES) and CXCL-8/IL-8 [18] that can further recruit inflammatory cells in areas of inflammation. CCL-2/MCP-1 (Figure 5e) and CCL-5/RANTES were indeed significantly up-regulated by the combination PHY906+CPT-11 compared to CPT-11 alone. Therefore, to compare the localization of immune cells in the tumors suggested by gene expression analysis, we analyzed the presence of macrophages in histologically prepared whole tumor cross-sections. As shown in Figure 5, tumors harvested 3 days post treatment had an increase in the number of F4/80 antibody positive cells in the CPT-11 and CPT-11+PHY906 treated tumors compared with the PBS control group indicating extensive macrophage infiltration. The comparison of CPT-11+PHY906 with CPT-11 alone revealed a strongly enhanced macrophage infiltration corroborating the transcriptional signatures. In contrast, PHY906 alone treatment demonstrated reduced macrophage infiltration compared with PBS alone confirming the anti-inflammatory effects of the herb when given alone. Thus, PHY906 carries opposite effects in the tumor microenvironment by reducing inflammation when given alone but enhancing acute pro-inflammatory processes within the tumor when given in combination with CPT-11.

### Validation by real-time qPCR of selected transcripts of relevance to this study

**Figure 6:**
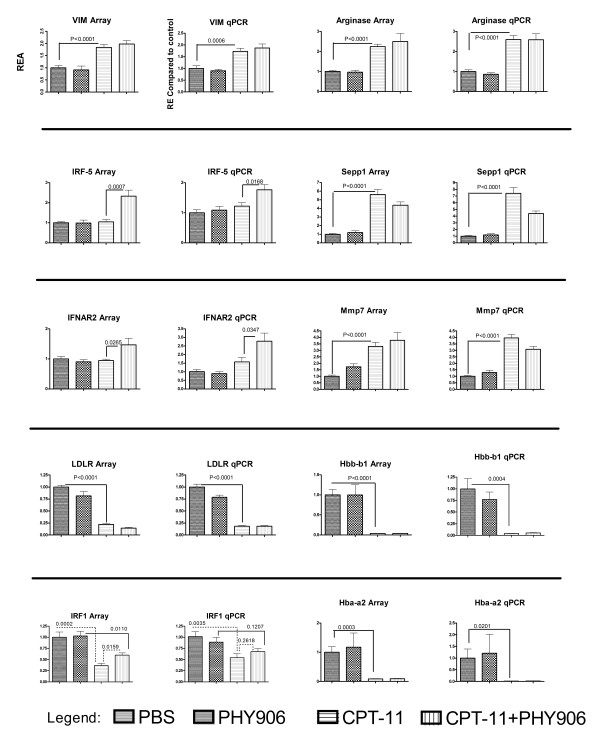
**Quantitative PCR validation of selected genes relevant to the study that were identified as differentially expressed according to transcriptional analysis among different treatment groups**. For each transcript, the first graph shows RNA array results followed by qPCR validation results. The Y-axis of the Array data graphs shows relative expression ratio of specific gene in samples relative to the mean of the same gene across PBS control group (REA-Relative Expression in Array). For qPCR validation, the data shown are of the relative expression (RE) of a gene of interest per actin molecule in sample compared to the averaged ratio of the gene of interest to actin for the entire control group (Y-axis). Statistics were Student t-tests (two tailed) performed by GraphaPad software. Error bars represent SD. The four graph bars on the X-axis for each graph represent the different treatment groups.

Several transcripts were selected for validation by qPCR (Figure 6). Although we have previously shown that qPCR may not necessarily be an optimal validation method for array data because it lacks endogenous reference controls and it is based on reference genes [19], it still provides an approximate corroboration of the information obtained with one platform. As shown here, data from array or qPCR in 10 transcripts relevant to the study and representing distinct behaviors in distinct treatment groups were compared side by side demonstrating a high level of comparability; of note is the behavior of IRF-5 which was confirmed to be up-regulated particularly in the PHY-906 with CPT-11 combination and IRF-1 which was significantly down-regulated in expression by CTP-11 (array p_2_-value = 0.0002 and qPCR p_2_-value = 0.0035) treatment. This phenomenon was partially reversed in the presence of PHY-906.

## Discussion

It has been suggested that conventional chemotherapy induces cancer cell elimination not only through direct cytostatic or cytotoxic mechanisms but also through modulation of host's immune cell function [20]. DNA damage caused by therapies that disrupt cell cycle has direct immune stimulatory effects. Tumors do not regress in immune depleted animals treated with doxorubicin given in combination with IL-12 and several other cytotoxic agents can induce powerful immune responses against cancer [20]. Although the effects of chemotherapeutic agents is currently believed to be due to the induction of cellular damage and subsequent activation of innate immune responses, no information is available about the transcriptional signatures within the tumor microenvironment that could support this hypothesis. A few experimental models suggest that chemotherapy acts by inducing the release of cytokines and chemokines such as CXCL-9/Monokine induced by IFN-γ (MIG), CXCL-10/IFN-γ -induced protein 10 kDa (IP-10), CCL-2/MCP-1, CCL-3/MIP-1α, CCL-5/RANTES, IL-6, IFN-γ and TNF [21,22]. This study assessed the effect of chemotherapy at the global transcript level.

The study focused on the early effects of treatment which are more likely to provide mechanistic information about the up-stream events leading to the observed phenomenology as we have previously shown in preclinical [23] or clinical models [24,25]. The results support the current view that chemotherapy acts, at least in part, by modulating immune responses (Figure 2c) resulting in enhancement of immune infiltrates (Figure 5c). Of note, CPT-11, modulated the high mobility group box-1 pathway, confirming the central role that it plays in the induction of anti-cancer activity by chemotherapeutics through activation of innate immunity [26,27]. The effects of CPT-11 on immune responses at the dose used in this study were, however, ambivalent with a balanced proportion of genes related to inflammatory processes being up-regulated or down-regulated over their baseline activation within the tumor microenvironment (Figure 2). CPT-11 effects were centered on the regulation of apoptosis by NF-*k*B including activation of genes related to mitochondrial fatty acid oxidation, sodium pump regulation such as the ATPase, Na+/K+ transporting, alpha 1 polypeptide (ATP1A1), the tumor necrosis factor receptor super-family member 12A (Fn14/TNFRF12A/TWEAKR) inducer of apoptosis, the tumor necrosis factor AIP3 interacting protein 2 (TNIP2), the inositol 1,4,5-trisphosphate 3-kinase B (ITPKB). These genes have predominantly pro-apoptotic effects, while genes with clear anti-apoptotic effects and related to cell growth such as lactate dehydrogenase A (LDHA) and the transferrin receptor (TFRC) were down-regulated; thus, it appears that CPT-11 counteracts the anti-apoptotic activity of NF-*k*B inducing apoptosis and alterations of cellular metabolism. In contrast, pro-inflammatory pathways downstream of NF-kB regulation were predominantly down-regulated at this early time point perhaps because the tissue damage induced by chemotherapy had not reached a sufficient intensity to induce immune activation (Figure 4a and 5c). The complex activation of NF-*k*B-dependent transcripts leading to apoptosis and/or inflammation deserves discussion. The anti-apoptotic effects of NF-*k*B are balanced in cancer by the tumor suppressor effects of IRF-1 and/or IRF-5 which directly inhibit its cyto-protective activity [14,28,29]. In particular, IRF-5 can sensitize tumors treated with CPT-11 to DNA damage-induced apoptosis and cell death [30]. In baseline conditions IRF-5 was found to be down-regulated in Colon 38 tumors compared to normal tissues while the expression of IRF-1 was neutral compared with other tissues (Figure 2a). We speculate that CPT-11 activates pro-apoptotic pathways by suppressing IRF-1 expression (Figure 5c) while leaving unaltered the constitutive down-regulation of IRF-5 in these tumors. The combined down-regulation of the two tumor suppressor genes may allow NF-kB to partially counteract apoptosis while the acute inflammatory process fostering cancer rejection is hampered by the down-regulation of the IRFs [31].

PHY906 was originally used to treat gastrointestinal symptoms including those related to the toxic effects of chemotherapy. Work from our group [4] demonstrates that PHY906 can revert intestinal damage caused by CPT-11 through the anti-inflammatory properties of the herb and the repopulation of intestinal progenitor cells. This study, did not address the complex effects that PHY906 has on the gut that will be addressed in the future, but focused on the early transcriptional changes observable at the tumor site. To address whether the effects of PHY906 were tissue specific, we compared the transcriptional changes at the tumor site with those in two normal organs characterized by high metabolic activity (liver) or immunologic functions (spleen). These organs were also exposed to systemic concentrations of orally administered PHY906 and/or CPT-11 (i.p.) different from those experienced by the front line exposure in the gut. Comparisons of the transcriptional profiles of the three tissues demonstrated clear baseline differences independent of any treatment which made cross comparisons of the various treatment effects among various tissues uninformative. Nevertheless, this approach clearly demonstrated that the effect of PHY906 and of chemotherapy with CPT-11 is exquisitely tissue-specific with very little overlap of pathways regulated by the various combinations in different tissues. This is not surprising since each tissue is enriched with different potential target cells that may react differently to CPT-11 or PHY906.

While tissues were not micro-dissected or single cell preparations were not obtained *ex vivo*, a general overview of the global changes induced by PHY906 could be obtained. In particular, we were interested in understanding how a herbal product with supposed anti-inflammatory properties could paradoxically increase the anti-tumor effects induced by chemotherapy; this concept is opposed to the current hypothesis of how the host immune system cooperates with the effects of chemotherapy [20]. Besides its immune modulatory effects on inflammation, PHY906 could have a direct impact on cancer cell survival by modulating apoptosis, autophagy or necrosis in cells undergoing exposure to chemotherapy. Although we have previously observed that PHY906 could inhibit tumor cell growth in culture (Additional file 4, **Data in S4**), the *in vitro *data could be misleading since not all the chemicals in PHY906 are necessarily absorbed into circulation and some that are absorbed will be metabolized *in vivo*. However, it is possible that some of the *in vitro *effects mediated by PHY906 on cancer cells may be retained. *In vivo*, PHY906 induced a predominantly downward modulation of transcription and down-regulation of the pro-inflammatory activity naturally present within the tumor. This anti-inflammatory effect was corroborated by the decreased amount of tissue macrophages seen by immunohistochemistry (Figure 5b).This is in line with its postulated functions in the gut [4]. Since no tumor regression was seen in response to PHY906 administered alone, it is unlikely that these effects have relevant bearing on tumor regression.

The surprising finding of this study emerged when we examined the effects of the combination of PHY906 plus CPT-11. Contrary to the anti-inflammatory properties observed when administered alone, PHY906 strongly counteracted the CPT-11 induced depression of inflammation through the IRF-mediated pathways. In particular, PHY906 counteracted the down-regulation of the master regulator of the pro-inflammatory switch and apoptosis IRF-1 [14,31,32]. Additionally, PHY906 enhanced the expression of IRF-5, another potent pro-inflammatory transcription factor associated with immune-mediated, tissue-specific rejection [33-36] as well as induction of apoptosis of CPT-11 treated cancers [30]. Thus, we speculate that PHY906, in line with other experimental models [20], enhances the anti-tumor properties of chemotherapy with CPT-11 by imparting a pro-inflammatory state that is not observed in the same CPT-11 naïve cancerous tissue. The reasons for the contradictory behavior remain to be ascertained, although it is likely that they involve a modulation of the balance between the anti-apoptotic and pro-inflammatory functions of NF-*k*B while, at the same time, inducing the expression of interferon stimulated genes with chemo-attractant properties such as CCL-2/MCP-1 and CCL-5/RANTES [29].

## Conclusions

In summary, this is the first study of the effects of a traditional Chinese compound PHY906 on tumors undergoing chemotherapy with CPT-11. The results demonstrated that 1) PHY906 and CPT-11 alone induce significant alterations in all tissues evaluated (tumor, liver and spleen); 2) PHY906 and CPT-11 alone generally induce repression of transcription in the tumors; 3) the effects of PHY906 are reverted in the presence of a chemotherapeutic agent such as CPT-11, with a high prevalence of transcripts being up-regulated; 4) PHY906 bears a general immune-suppressive effect in tumors when given alone but, when given in combination, it results in a relative pro-inflammatory pro-apoptotic effect; 5) the pro-inflammatory, pro-apoptotic effects of PHY906 when given in combination are exquisitely tumor-specific as they are not observed in the gut as we recently described [4] nor in other normal tissues such as liver or spleen as observed in this study. It should be emphasized that this striking dichotomy in the behavior of this herbal product suggest its usefulness to enhance the therapeutic window for chemotherapeutics as it can decrease toxicity in normal tissues while at the same time promoting cell death within the tumor microenvironment.

## Competing interests

Y.-C.C. is a scientific founder of and has equity interest in PhytoCeutica Inc., a company that develops traditional Chinese medicine into drugs for the treatment of cancer and that licenses PHY906 from Yale University. Z.J. owns stock in PhytoCeutica Inc. Yale University holds a patent on the herbal composition PHY906 and its use in chemotherapy.

## Authors' contributions

EW, SB, FMM, JC, YCC: designed research, performed research, contributed to new reagents/analytic tools, analyzed and interpreted data, wrote paper; JW: designed research, contributed to new reagents/analytic tools; CQ, YM: analyzed and interpreted data; WL, FG, ZJ: performed research, analyzed and interpreted data; DB, YZ, DFS: analyzed and interpreted data, wrote paper. All authors have read and approved the manuscript.

## Pre-publication history

The pre-publication history for this paper can be accessed here:

http://www.biomedcentral.com/1755-8794/4/38/prepub

## Supplementary Material

Additional file 1 **Table S1**. qPCR mouse primers used. Click here for file

Additional file 2 **Table S2**. Summary of differentially expressed genes among treatment groups in different tissues. Click here for file

Additional file 3 **Figure S3**. Stacked bar chart summarizing the 15 most affected canonical pathways according to IPA based on genes with annotated function differentially expressed (t-test cutoff p-value < 0.05, *pt *test p-value < 0.001) in tumor biopsies between PBS control group and the treatment groups: (**a**) PHY906 (**b**) CPT-11 (**c**) PHY906+CPT-11. Finally, differences between PHY906+CPT-11 compared to CPT-11 alone are shown in (**d**). Click here for file

Additional file 4 **Data S4**. Cytotoxicity of PHY906 on HepG2 after three days exposure. Click here for file
