# Supplementary material for: Interaction of a traditional Chinese Medicine (PHY906) and CPT-11 on the inflammatory process in the tumor microenvironment
Source: BMC Med Genomics. 2011 May 11;4:38. doi: 10.1186/1755-8794-4-38 (PMC3117677; doi:10.1186/1755-8794-4-38)
Supplement: Additional file 1 — Table S1. qPCR mouse primers used. [file 1755-8794-4-38-S1.DOC]

**Table S1.qPCR mouse primers used.**

| **Gene** | **Forward** | **Reverse** | **RefSeq ID** |
| --- | --- | --- | --- |
| **SEPP1** | AGCTCTGCTTGTTACAAAGCC | CAGGTCTTCCAATCTGGATGC | NM_009155 |
| **MMP7** | CTGCCACTGTCCCAGGAAG | GGGAGAGTTTTCCAGTCATGG | NM_010810 |
| **Hba-a2** | TGCATGCCCACAAGCTGCGT | GCAGGCTTCTTCCTACTCAGGC | NM_001083955.1 |
| **Hbb-b1** | GCACCTGACTGATGCTGAGAA | TTCATCGGCGTTCACCTTTCC | NM_008220 |
| **B-Actin** | CGTGGGCCGCCCTAGGCACCA | TTGGCCTTAGGGTTCAGGGGGG | NM_007393 |
| **LDLr** | GGTTCCTGTCCATCTTCTTCC | TCTTCAGCCGCCAGTTCC | NM 010700.2 |
| **Arginase1** | TGAGAGACCACGGGGACCTG | GCACCACACTGACTCTTCCATTC | NM_007482.3 |
| **IRF5** | ACACTGAAGGGGTGGATGAGGC | AAGGAATAGGGTGCGTTGGGAGGC | NM_012057.3 |
| **Vimentin** | GCTGGAAGGCGAGGAGAGCA | TGGGTGTCAACCAGAGGAAGTGA | NM_011701.4 |
| **IRF1 Transcript 2** | GGCTCGCTGCCTTGACTGGCT | TGTTGATGTCCCAGCCGTGCTTAG | NM_001159396.1 |
| **IFNAR2**  **(Interferon alpha/beta receptor 2 isoform a**) | TGGTAGTGATGGTTTTCGTGAGCAC | CAGAGGCTTGCCCGTCAGTCC | NM_010509.2 |
