# Supplementary material for: Interaction of a traditional Chinese Medicine (PHY906) and CPT-11 on the inflammatory process in the tumor microenvironment
Source: BMC Med Genomics. 2011 May 11;4:38. doi: 10.1186/1755-8794-4-38 (PMC3117677; doi:10.1186/1755-8794-4-38)
Supplement: Additional file 2 — Table S2. Summary of differentially expressed genes among treatment groups in different tissues. [file 1755-8794-4-38-S2.DOC]

|  | **Test** | **Number of genes < 0.001 and**  **(< 0.05 total/annotated)*** | ***Pt*  p-value**  **for < 0.001 cutoff^†^** |
| --- | --- | --- | --- |
| **Tumor Microenvironment** |  |  |  |
| **Tumor vs Spleen and Liver** |  |  |  |
| **Tumor PBS vs Spleen+Liver PBS** | t test | (7,856/7,348) | **< 0.001** |
| **Tumor PHY906 vs Spleen+Liver PBS** | t test | (6,890/6,485) | **< 0.001** |
| **Tumor CPT-11 vs Spleen+Liver PBS** | t test | (7,125/6,657) | **< 0.001** |
| **Tumor CPT-11+PHY906 vs Spleen+Liver PBS** | t test | (7,721/7,015) | **< 0.001** |
|  |  |  |  |
| **Four way** | F test | **1,132** | **< 0.001** |
| **PBS vs PHY906** | t test | **117/113** (1,759/1,664) | **< 0.001** |
| **PBS vs CPT-11** | t test | **570/557** (2,630/2,513) | **< 0.001** |
| **PBS vs CPT-11+PHY906** | t test | **798/774** (3,347/3,190 ) | **< 0.001** |
| **CPT-11 vs CPT-11+PHY906** | t test | **93/91** (1,556/1,477 ) | **< 0.001** |
| ***(number of samples; PBS = 10; PHY906 = 9;* CPT-11 *= 10;* CPT-11 *+PHY906 = 9; Total 38)*** | | | |
|  |  |  |  |
| **Spleen** |  |  |  |
| **Four way** | F test | **2,689** | **< 0.001** |
| **PBS vs PHY906** | t test | **1,000** (3,702/3,450) | **< 0.001** |
| **PBS vs CPT-11** | t test | **1,202** (4,235/3,956) | **< 0.001** |
| **PBS vs CPT-11+PHY906** | t test | **2,192** (5,576/5,181) | **< 0.001** |
| **CPT-11 vs CPT-11+PHY906** | t test | **92** (1,824/1,682) | **0.009** |
| ***(number of samples; PBS = 10; PHY906 = 10;* CPT-11 *= 8;* CPT-11 *+PHY906 = 10; Total 38)*** | | | |
|  |  |  |  |
| **Liver** |  |  |  |
| **Four way** | F test | **560** | **< 0.001** |
| **PBS vs PHY906** | t test | **168** (2,116/1,932) | **< 0.001** |
| **PBS vs CPT-11** | t test | **432** (2,961/2,678) | **< 0.001** |
| **PBS vs CPT-11+PHY906** | t test | **575** (3,052/2769) | **< 0.001** |
| **CPT-11 vs CPT-11+PHY906** | t test | **172** (1,601/1,436) | **0.019** |
| ***(number of samples; PBS = 10; PHY906 = 8;* CPT-11 *= 9;* CPT-11 *+PHY906 = 8; Total 35)*** | | | |

**Table S2.** Summary of differentially expressed genes among treatment groups in different tissues.

* Cutoff p-value < 0.05 was used to select genes for IPA. In parentheses number of genes identified at 0.05 level and of those with annotated function that could be used for IPA; ^†^although the *pt* test p-value is shown for the <0.001 analyses, identical results were obtained for the lower stringency analyses

Note. *Pt* test: permutation test
