# Supplementary material for: Interaction of a traditional Chinese Medicine (PHY906) and CPT-11 on the inflammatory process in the tumor microenvironment
Source: BMC Med Genomics. 2011 May 11;4:38. doi: 10.1186/1755-8794-4-38 (PMC3117677; doi:10.1186/1755-8794-4-38)
Supplement: Additional file 3 — Figure S3. Stacked bar chart summarizing the 15 most affected canonical pathways according to IPA based on genes with annotated function differentially expressed (t-test cutoff p-value < 0.05, pt test p-value < 0.001) in tumor biopsies between PBS control group and the treatment groups: (a) PHY906 (b) CPT-11 (c) PHY906+CPT-11. Finally, differences between PHY906+CPT-11 compared to CPT-11 alone are shown in (d). [file 1755-8794-4-38-S3.PPT]

## Slide 1
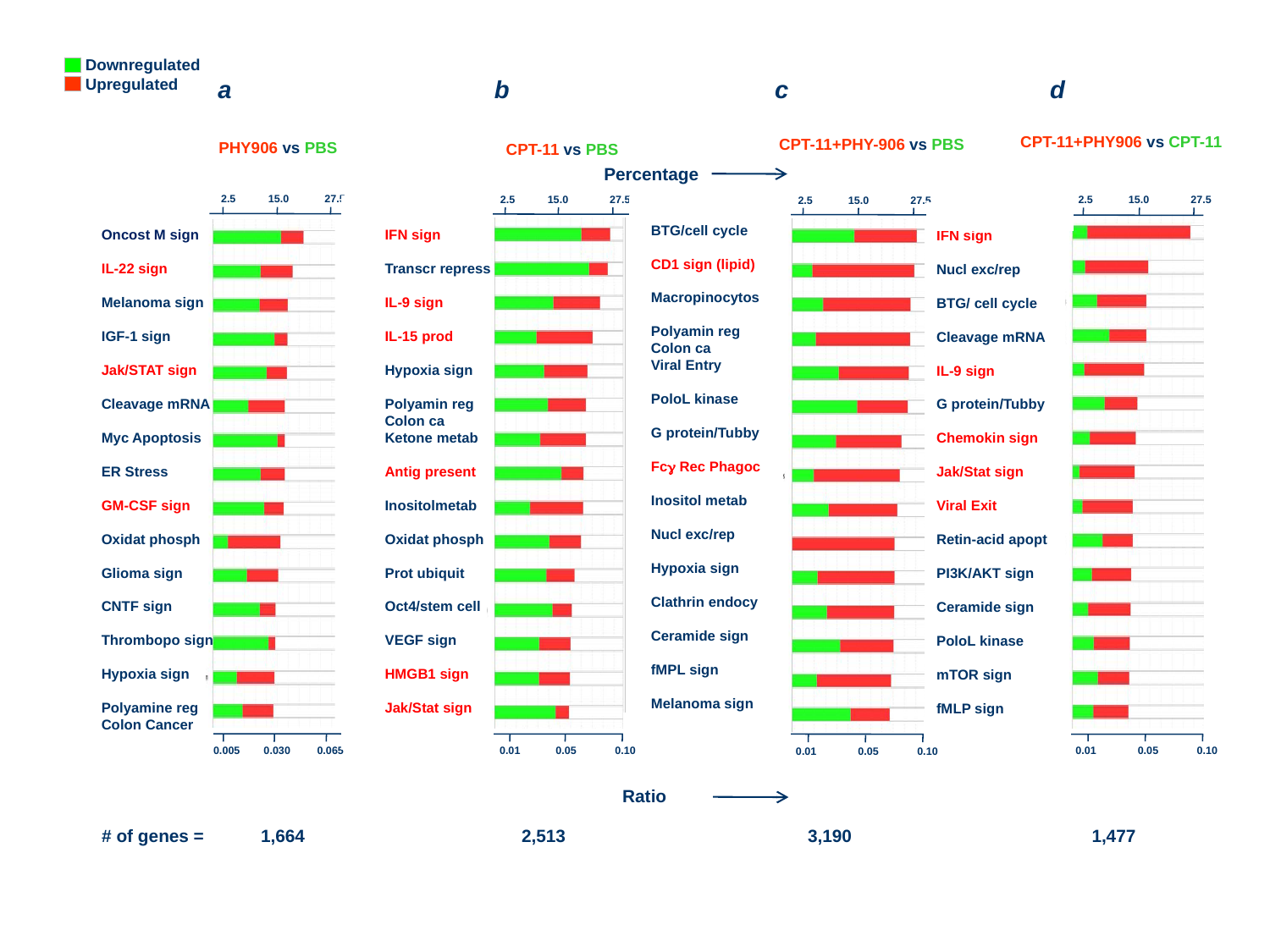

Downregulated
Upregulated
a
b
c
d
CPT-11+PHY906 vs CPT-11
CPT-11+PHY-906 vs PBS
PHY906 vs PBS
CPT-11 vs PBS
BTG/cell cycle
CD1 sign (lipid)
Macropinocytos
Polyamin reg
Colon ca
Viral Entry
PoloL kinase
G protein/Tubby
Fcγ Rec Phagoc
Inositol metab
Nucl exc/rep
Hypoxia sign
Clathrin endocy
Ceramide sign
fMPL sign
Melanoma sign
Oncost M sign
IL-22 sign
Melanoma sign
IGF-1 sign
Jak/STAT sign
Cleavage mRNA
Myc Apoptosis
ER Stress
GM-CSF sign
Oxidat phosph
Glioma sign
CNTF sign
Thrombopo sign
Hypoxia sign
Polyamine reg
Colon Cancer
IFN sign
Transcr repress
IL-9 sign
IL-15 prod
Hypoxia sign
Polyamin reg
Colon ca
Ketone metab
Antig present
Inositolmetab
Oxidat phosph
Prot ubiquit
Oct4/stem cell
VEGF sign
HMGB1 sign
Jak/Stat sign
IFN sign
Nucl exc/rep
BTG/ cell cycle
Cleavage mRNA
IL-9 sign
G protein/Tubby
Chemokin sign
Jak/Stat sign
Viral Exit
Retin-acid apopt
PI3K/AKT sign
Ceramide sign
PoloL kinase
mTOR sign
fMLP sign
# of genes =	1,664	2,513	3,190		1,477
Percentage
 2.5 15.0 27.5
 2.5 15.0 27.5
 2.5 15.0 27.5
 2.5 15.0 27.5
 0.01 0.05 0.10
 0.005 0.030 0.065
 0.01 0.05 0.10
 0.01 0.05 0.10
Ratio
