# Supplementary material for: Interaction of a traditional Chinese Medicine (PHY906) and CPT-11 on the inflammatory process in the tumor microenvironment
Source: BMC Med Genomics. 2011 May 11;4:38. doi: 10.1186/1755-8794-4-38 (PMC3117677; doi:10.1186/1755-8794-4-38)
Supplement: Additional file 4 — Data S4. Cytotoxicity of PHY906 on HepG2 after three days exposure. [file 1755-8794-4-38-S4.DOC]

**Data S4. Cytotoxicity of PHY906 on HepG2 after three days exposure: IC_50_ = .85 mg/ml (+/- .18).**
Concentration was based on the total dry PHY906 product weight. PHY906 was extracted in water for 30 minutes at 80°C. Cell growth inhibition was measured using the methylene blue uptake assay. The HepG2 cell line was routinely grown in MEME media, supplemented with 10% fetal bovine serum (FBS) and 100 ug/ml kanamycin. Cells were incubated at 37^o^ C in a humidified atmosphere of 5% CO_2_: 95% air. Cancer cells (1x10^4^) were seeded into a 24-well plate in either 1 ml of MEME medium with 10% FBS and 100 ug/ml kanamycin on day 0. The freshly prepared and sterilized PHY906 extract was added to cells on day 1, at various concentrations, and incubated at 37^o^C for 3 days. The medium was then removed, and the cell layer was stained for 30 min with 0.3 ml of 0.5% (w/v) methylene blue solution (in 50% ethanol). The plates were washed 3 times with tap water, dried, and the cell layer was lysed with 1 ml of 1% Sarkosyl solution (in PBS). The lysates solution was read on an Elx800 kinetic microplate reader (Bio-Tek Instruments, Inc.) at 595 nm.
